# ZNF32 histidine 179 and 183 single-site and double-site mutations promote nuclear speckle formation but differentially regulate the proliferation of breast cancer cells

**DOI:** 10.3389/fcell.2025.1490231

**Published:** 2025-02-19

**Authors:** Chaosong Zhong, Dingshuang Chen, Fei Wang, Junqi Wang, Ruiwen Li, Yanyan Li, Di Gong

**Affiliations:** ^1^ Key Laboratory of Qinghai-Tibetan Plateau Animal Genetic Resource Reservation and Utilization, Ministry of Education, Southwest Minzu University, Chengdu, China; ^2^ College of Animal and Veterinary Sciences, Southwest Minzu University, Chengdu, China; ^3^ Chengdu Women’s and Children’s Central Hospital, School of Medicine, University of Electronic Science and Technology of China, Chengdu, China; ^4^ School of Basic Medical Science, Chengdu University, Chengdu, China

**Keywords:** ZNF32, breast cancer, nuclear speckles, mutation, proliferation

## Abstract

Studies have shown that histidine 179A and 183A (H^179, 183^A) of the ZNF32 protein exhibit point-like nuclear speckles, but the causes of such speckle formation and their effects on breast cancer cells remain unknown. In this study, we prepared breast cancer cells containing ZNF32 H^179, 183^A, H^179^A, and H^183^A and observed nuclear speckles in all three cell types. Transcriptome analysis showed that these nuclear speckles may be related to changes in the activities of the cell growth factor and RNA polymerase II transcription factor. Comprehensive transcriptomics and metabolomics analyses showed that the formation of ZNF32 nuclear speckles was accompanied by changes in choline metabolism. Both *in vivo* and *in vitro* experiments suggested that ZNF32 H^179^A and H^183^A but not H^179, 183^A could promote breast cancer cell proliferations. We then explored and verified the differentially expressed genes through RNA-seq and RT-qPCR to explain the different proliferation abilities of these mutations. The dual luciferase reporter gene assay confirmed that ZNF32 H^179^A and H^183^A could transcriptionally activate *ISY1-RAB43* and *UPK3BL1* while inhibiting the transcription of *SNX22*; this is attributable to the fact that these mutations cause different zinc finger structure changes in ZNF32. The present study deepens the understanding of ZNF32 mutations with respect to nuclear speckle formation and their roles in the proliferation of breast cancer cells.

## 1 Introduction

Cancer refers to a large group of diseases resulting from the accumulation of mutations, chromosomal instabilities, and epigenetic changes that collectively impair the growth and death systems of cells (Hsu and Sabatini, 2008; Jones and Thompson, 2009). Uncontrolled realization of replication immortality is one of the basic hallmarks of cancerous cells (Currie et al., 2013). Of all the known forms of cancer, breast cancer is the most common among women, and environmental deterioration as well as lifestyle defects are known to especially increase the incidence of this type of cancer (Zhu et al., 2023; De Cicco et al., 2019). The heterogeneity, variable subtypes, and diversity of signaling pathways of breast cancer greatly increase treatment difficulty (Roulot et al., 2016; Anderson et al., 2014; Yu et al., 2019). Therefore, identification of novel therapeutic targets for breast cancer and its tumor growth mechanisms are important and needed urgently.

The Cys2-His2 zinc finger (C_2_H_2_-ZF) proteins represent the largest class of putative human transcription factors (Lander et al., 2001). Zinc finger proteins play important roles in various cellular functions, including cell proliferation, differentiation, and apoptosis, through multiple zinc fingers and other functional modules (Schmitges et al., 2016; Weirauch and Hughes, 2011). Zinc finger protein 32 (ZNF32) is a confirmed nuclear protein that acts as a transcription factor to regulate the transcription of target genes *GPER* and *C1QBP* to affect stem-cell-like characteristics as well as cancer cell apoptosis, respectively (Li et al., 2018; Li et al., 2015). According to our previous studies, since ZNF32 does not contain a classical nuclear localization signal, we found that the nuclear localization sequence of ZNF32 could be between 170 and 228 amino acids (Aa) (Wei et al., 2016). Notably, among the previously constructed ZNF32 mutants, we found that the histidine 179 and 183 positions of ZNF32 show obvious nuclear speckles (NSs) in 293T cells. These results suggest that mutations at positions Aa179 and Aa183 of ZNF32 play important but neglected roles in NS formation and breast cancer progression.

NSs are also known as interchromatin granules and are small membraneless organelles located in the nucleus (Zhu and Brangwynne, 2015). NSs were first observed in 1910 under a light microscope. The term “speckles” was first used by J. Swanson Baker in 1961 to describe these components, which typically appear as 20–50 granules of varying sizes in most mammalian cells and are generally spherical with diameters of the order of several nanometers (Ilık and Aktaş, 2022). These highly dynamic condensates are rich in mRNA splicing factors, mRNA export proteins, transcriptional regulators (Saitoh et al., 2004), non-coding RNAs (Tripathi et al., 2010), and various other regulatory proteins, as well as DNA repair factors (Campalans et al., 2007; Wong et al., 2013). Most nuclear-membrane-free organelles are rich in proteins that specify their functions, such as ribosome assembly, splice assembly, and histone mRNA processing (Arias Escayola and Neugebauer, 2018). NSs are believed to play major roles in regulating the availability of splicing factors at the transcriptional sites and are associated with various dysfunction-related diseases, including cancer and viral diseases. However, current research on NSs is still limited, and there are gaps in the exploration of reasons for the formation of NSs and related functional mechanisms (Spector and Lamond, 2011).

In the present study, we successfully induced ZNF32 histidine 179 and 183 double-site (H^179, 183^A) and single-site (H^179^A, H^183^A) mutations in breast cancer cell lines and observed the appearance of NSs in the cells. We detected changes in the genes and metabolites related to NS formation in breast cancer through RNA-seq and metabolome sequencing. We also evaluated the effects of different ZNF32 mutants on tumor formation and growth processes in mouse models. *In vivo* and *in vitro* experiments were conducted to confirm that ZNF32 histidine 179 and 183 single-site mutations (H^179^A, H^183^A) but not double-site mutation (H^179, 183^A) could promote the proliferation of breast cancer cells. In addition, we screened four differentially expressed genes (DEGs) via RNA-seq to explain the strong proliferation abilities of the cancer cells in the single-site mutation groups. The dual luciferase reporter gene assay confirmed that ZNF32 H^179^A and H^183^A transcriptionally activate *ISY1-RAB43* and *UPK3BL1* as well as inhibit the transcription of *SNX22*. Our study thus deepens the understanding of the functions of ZNF32 mutants as well as NSs in breast cancer cells while providing a basis for finding new treatments for breast cancer.

## 2 Results

### 2.1 Histidine 179 and 183 double-site and single-site mutations of ZNF32 form NSs in breast cancer cells

**FIGURE 1:**
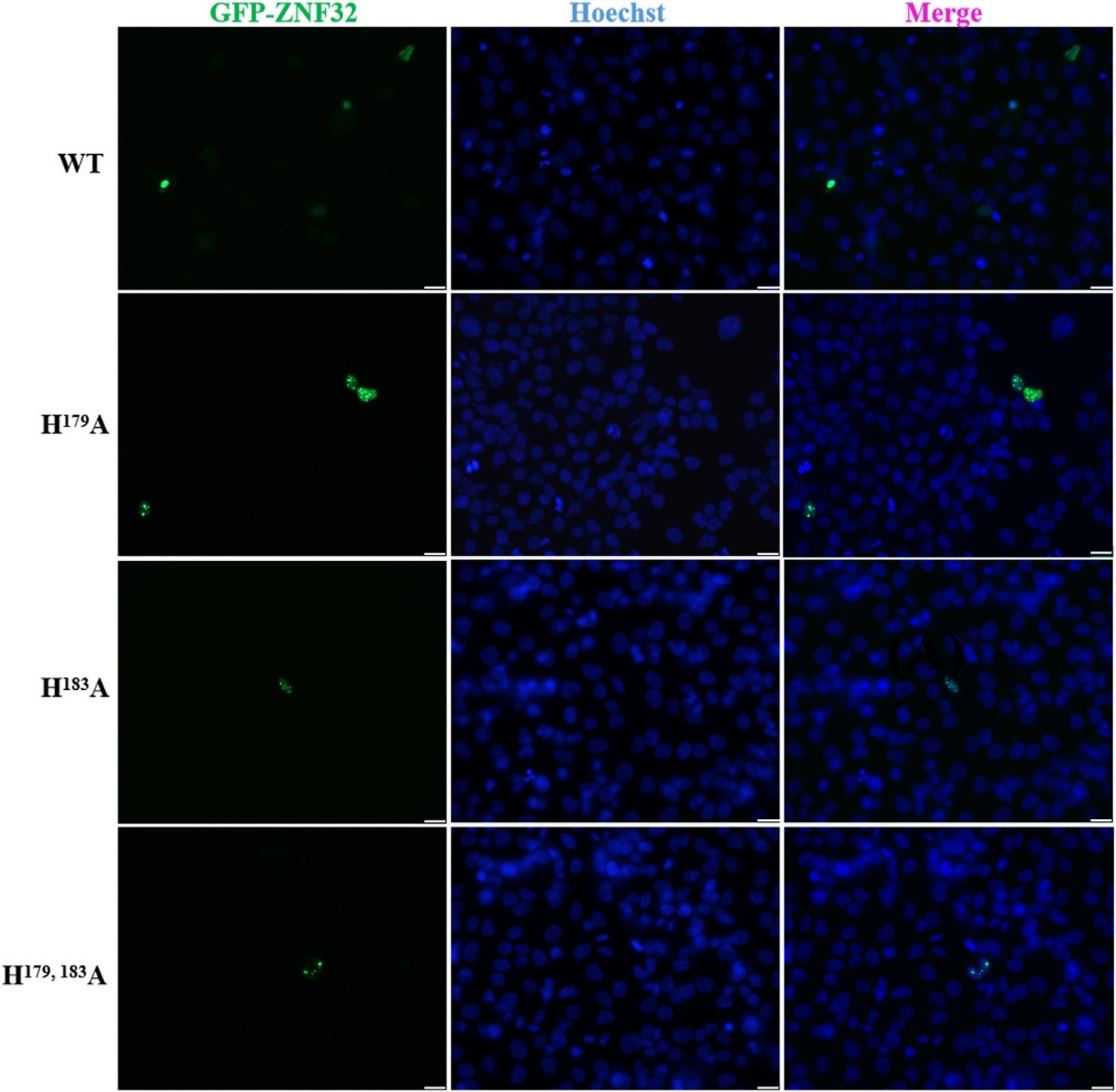
Subcellular localizations of ZNF32 mutants (H^179^A, H^183^A, and H^179, 183^A) in ZR-75-30 breast cancer cells. The recombinant proteins are shown with green fluorescence (GFP), and the cell nuclei are shown in blue (Hoechst). Scale bar = 20 μm.

Previous research results have shown that the nuclear localization sequence of ZNF32 may be located between Aa170 and Aa228. Among the previously constructed ZNF32 mutants, we found that the double-site mutations H^179, 183^A will form NSs in 293T cells (Wei et al., 2016). To study the exact roles of ZNF32 NSs caused by mutations of histidine 179 and 183 in breast cancer cells, we first constructed green fluorescence protein (GFP) fusion expression plasmids with H^179^A, H^183^A, and H^179, 183^A of ZNF32 for transfection to breast cancer cells using ZR-75-30 breast cancer cells overexpressing ZNF32 (wild-type or WT) as the control. As expected, the three mutant cells (H^179^A, H^183^A, H^179, 183^A) all showed ZNF32 NSs while the WT cells did not (Figure 1). In addition, we performed the same experimental verifications on two other breast cancer cell lines, namely MCF-7 and MDA-MB-231, and the results were consistent with those obtained with the ZR75-30 line (Supplementary Figure S1). Therefore, we speculate that ZNF32 H^179^A, H^183^A, H^179, 183^A can lead to formation of NSs in breast cancer cells.

### 2.2 RNA-seq analysis reveals that ZNF32 NS formation is related to RNA polymerase II transcriptional activity and may affect breast cancer cell growth

**FIGURE 2:**
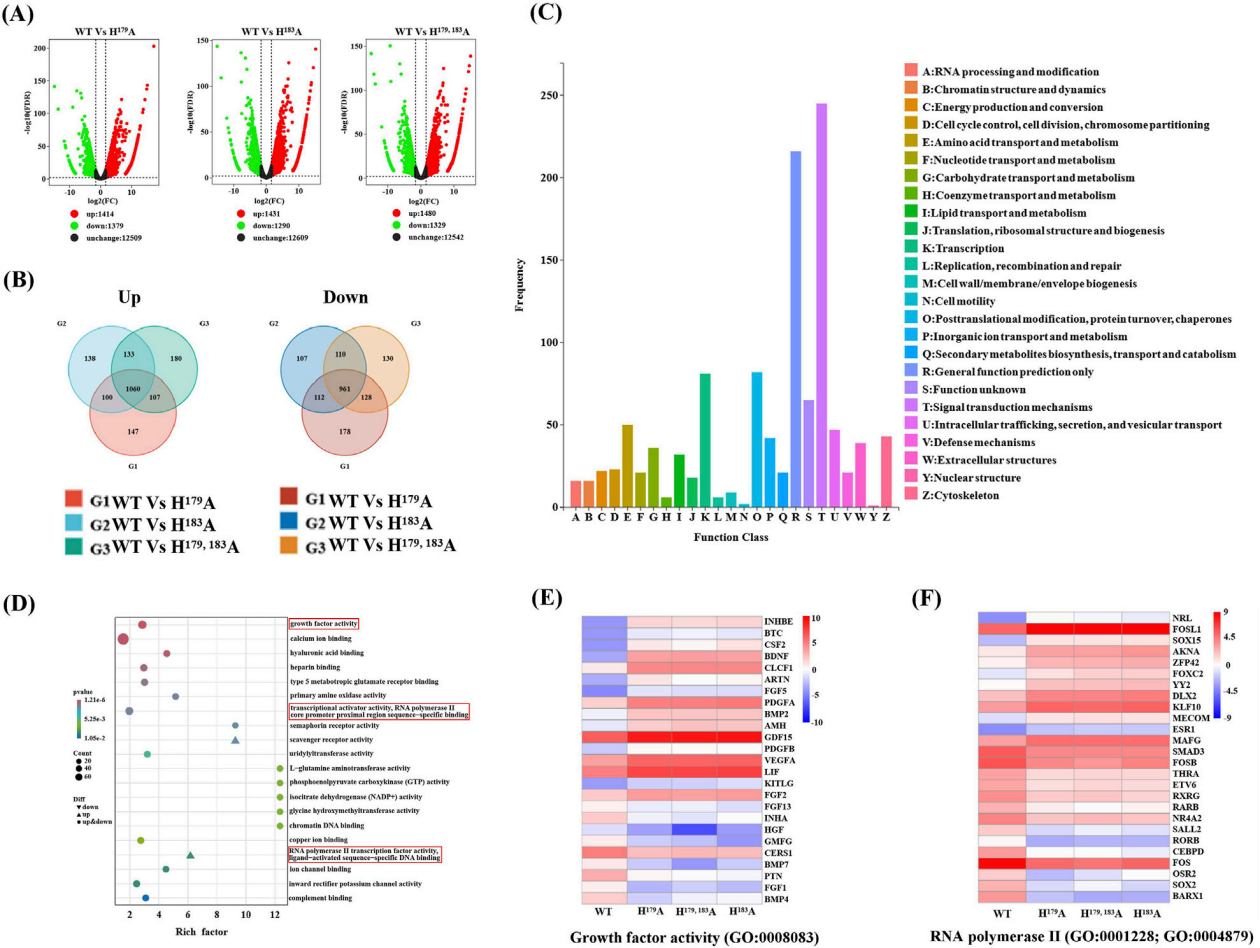
RNA-seq analyses of the differentially expressed genes (DEGs). **(A)** Volcano map showing the distribution of the DEGs, where the abscissa represents log2(fold change) and ordinate represents -log10(FDR). Black indicates genes with non-significant differences, while red and green indicate genes with significant differences; the red color represents upregulated genes, green color represents downregulated genes, and black color represents unchanged genes. **(B)** Venn diagrams of the upregulated and downregulated groups of DEGs according to study requirements, showing the numbers of DEGs unique to each comparison group and common DEGs among the comparison groups. **(C)** KEGG orthology (KOG) classification, where the horizontal axis is the classification content of the KOG database and the vertical axis is the number of genes annotated to the corresponding classification. **(D)** Gene ontology (GO) function enrichment of the DEGs, where the molecular functions are annotated according to their degrees of difference. **(E)** Clustering heatmap of DEGs associated with growth factor activity (GO:0008083). **(F)** Clustering heatmap of DEGs associated with RNA polymerase II transcription activity (GO:0001228; GO:0004879).

To better understand the causes of ZNF32 NS formation, lentiviral vectors were used to construct breast cancer cell lines with stable mutations at these sites, and the increases in ZNF32 expressions compared with endogenous levels were detected by reverse transcription quantitative real-time polymerase chain reaction (RT-qPCR) (Supplementary Figure S2). RNA-seq was conducted to analyze the DEGs associated with NS formation. ZNF32 H^179^A, H^183^A, and H^179, 183^A were compared with the control group, and three groups of DEGs were obtained after screening. Accordingly, a total of 1,414 upregulated and 1,379 downregulated DEGs were screened in the WT vs. H^179^A, a total of 1,431 upregulated and 1,290 downregulated DEGs were screened in the WT vs. H^183^A, and a total of 1,480 upregulated and 1,329 downregulated DEGs were screened in the WT vs. H^179, 183^A (Figure 2A). To obtain DEGs with the same expression trends, we found the intersection of the three groups of upregulated DEGs and obtained 1060 genes. Similarly, the intersection of the three groups of downregulated DEGs showed 961 genes (Figure 2B). Thus, a total of 2021 DEGs with the same up-down-regulation trends were obtained. The details of these DEGs and their enrichment in each database are presented in Supplementary Table S1. The Kyoto Encyclopedia of Genes and Genomes orthology (KOG) enrichment analysis was performed on these DEGs, and it was found that the genes were mainly enriched in terms of functional classifications, such as signal transduction mechanisms, posttranslational modifications, transcription, as well as amino acid transport and metabolism (Figure 2C). Gene ontology (GO) enrichment analysis showed that the molecular functions of the DEGs mainly included growth factor activity, calcium binding, ATPase activity, transcription activator activity, ion-channel binding, and RNA polymerase II transcription factor activity (Figure 2D). Therefore, NS formation may affect cell proliferation, and the DEGs enriched for growth factor activity (GO:0008083) are shown in (Figure 2E). It has been reported that the appearance of NSs is related to the transcriptional activity of RNA polymerase II (Wei et al., 1999, Bregman et al., 1995). Therefore, we speculate that the DEGs related to RNA polymerase II (GO:0001228; GO:0004879) may play important roles in the formation of NSs, as shown in (Figure 2F). In addition, the DEGs in the KEGG pathway showed that the most significantly enriched pathways were those of glycine, serine, and threonine metabolisms, including the pathways related to cancer, MAPK, PI3K-Akt, Rapl, and RAS signaling, which are closely related to the proliferation of cancer cells. Together, these indicate that ZNF32 NS formation maybe related to the transcriptional activity of RNA polymerase II and growth factor activity and that these may affect the growth of breast cancer cells.

### 2.3 Joint transcriptomics and metabolomics analysis reveals that ZNF32 NS formation is accompanied by changes in multiple signaling pathways

**FIGURE 3:**
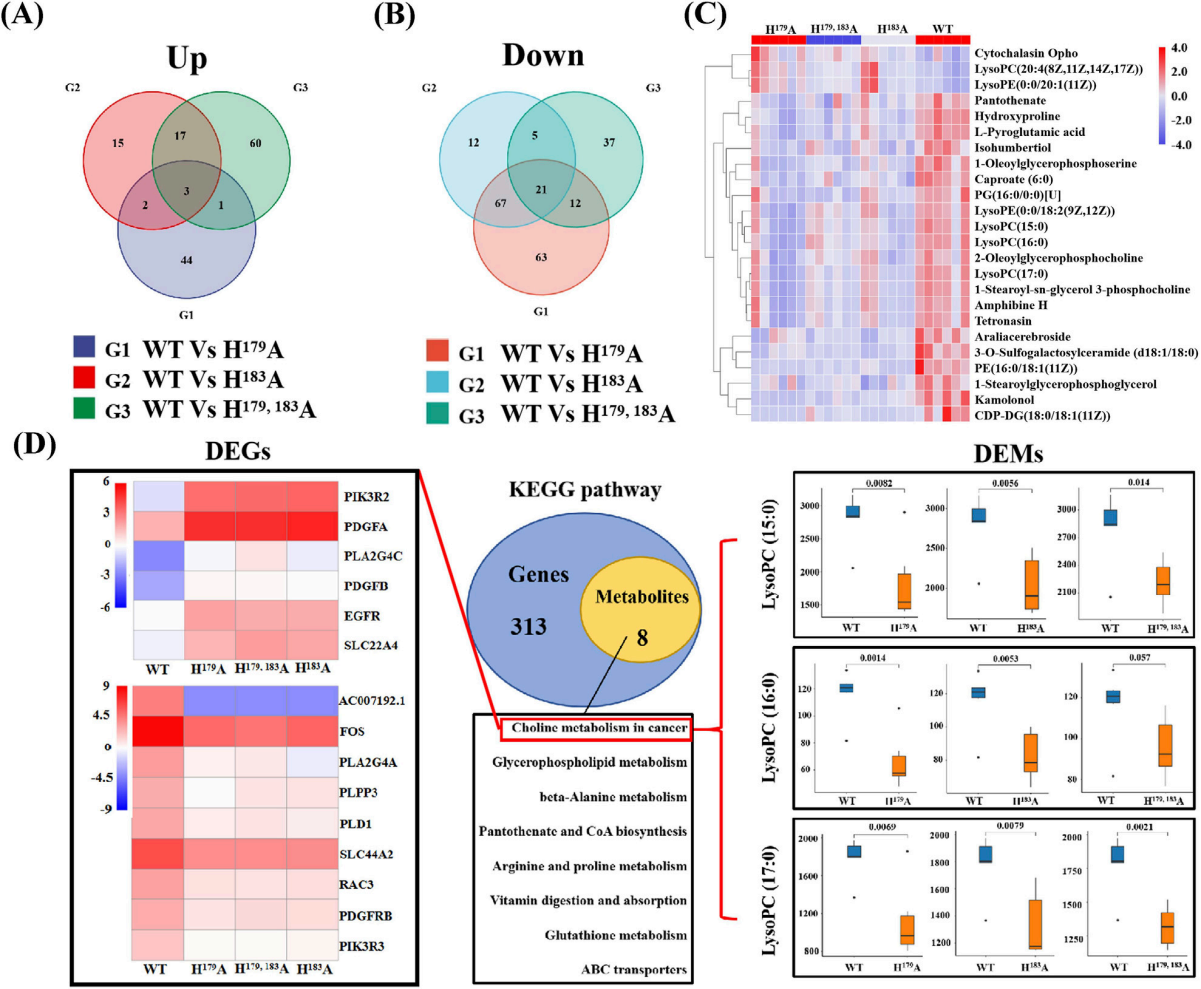
Joint transcriptome and metabolomics analysis. Venn diagrams of the **(A)** upregulated and **(B)** downregulated differentially expressed metabolites (DEMs). **(C)** Clustering heatmap showing expression differences of the DEMs in different groups. **(D)** Venn diagram of the differential gene and differential metabolite pathways. The number of common pathways was obtained by comparing the pathways of the genes in the transcriptional group and pathways of the metabolites in the metabolic group. On the left are the DEGs enriched in the choline metabolic pathway showing the upregulated and downregulated heatmaps; on the right are the DEMs enriched in the choline metabolic pathway and the expression level comparison of the corresponding groups.

Metabolomics was used to further study the functions of ZNF32 H^179^A, H^183^A, and H^179, 183^A that cause NSs. We compared the ZNF32 H^179^A, H^183^A, and H^179, 183^A groups with the control WT group and screened out the differentially expressed metabolites (DEMs). To obtain DEMs with the same expression trends, we considered the intersections of the three groups to obtain three common upregulated and 21 common downregulated DEMs (Figure 3A, B). We plotted the set of screened differential metabolites as a cluster heatmap for display, and we believe that these 24 DEMs are likely to be related to the functions of the NSs (Figure 3C). The details of these DEMs and their enrichment in each database are presented in Supplementary Table S2. We also conducted a joint transcriptomics and metabolomics analysis to compare the KEGG pathways enriched by the 2021 DEGs obtained from the transcriptome analysis with those enriched by the 24 DEMs from the metabolome analysis. The DEMs were enriched in eight pathways and overlapped with 321 pathways enriched in terms of DEGs (Figure 3D). These eight signal pathways include choline metabolism in cancer, glycerophospholipid metabolism, beta-alanine metabolism, pantothenate and CoA biosyntheses, and arginine and proline metabolisms. A total of 15 DEGs and 3 DEMs were enriched in the choline metabolism pathway. Among these, the upregulated DEGs are *PIK3R2, PDGFA, PLA2G4C, PDGFB, EGFR,* and *SLC22A4*, while the downregulated DEGs are *AC007192, FOS, PLA2G4A, PLPP3, PLD1, SLC44A2, RAC3, PDGFRB,* and *PIK3R3*. The expression levels of the three DEMs, namely LysoPC (15:0), LysoPC (16:0), and LysoPC (17:0), were all downregulated. These results indicate that the formation of ZNF32 NSs is accompanied by changes in choline metabolism in cancer.

### 2.4 ZNF32 H^179^A, H^183^A, and H^179, 183^A cause NSs and different proliferation effects in breast cancer cells

**FIGURE 4:**
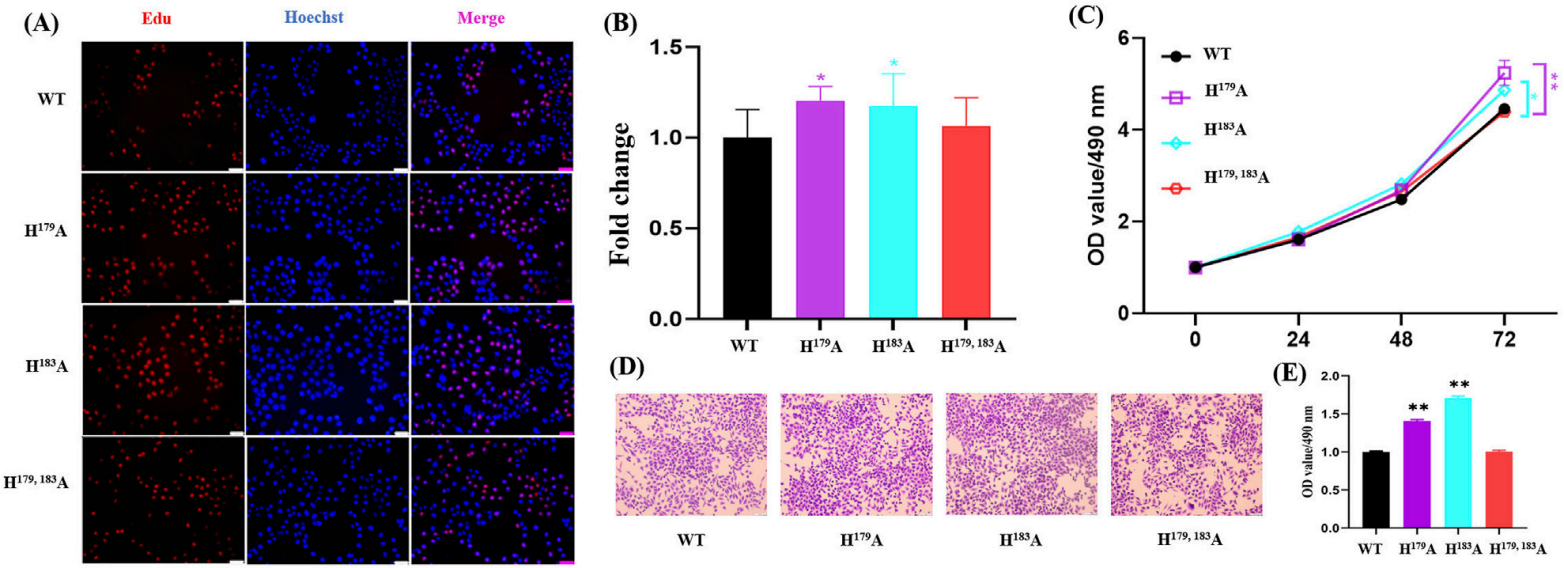
Effects of ZNF32 (H^179^A, H^183^A, and H^179, 183^A) on cell proliferation showing **(A)** results of EdU staining, **(B)** ratio of EdU-positive cells, and **(C)** viability analysis. MTT assay was performed according to manufacturer protocols. Absorbances of the samples were measured at 490 nm. **(D, E)** Crystal violet staining analysis of the cells. Wild-type (WT) and mutant cells of the ZNF32 locus were cultured for 3 weeks and stained with crystal violet staining solution; semi-quantification was used to examine the cell numbers.

The results of the omics analyses indicate that NS formation is related to the transcriptional activity of RNA polymerase II and also causes changes in several cancer-related metabolic pathways. Studies have shown that abnormal choline metabolism is related to the growth, differentiation, invasion, and metastasis of cancer cells (Glunde et al., 2011; Cao et al., 2016). Previous works have reported that RNA polymerase II activity is associated with cell proliferation (Vervoort et al., 2022; Huang and Ji, 2023; Giakountis et al., 2017). Hence, we hypothesized that ZNF32 H^179^A, H^183^A, and H^179, 183^A causing NSs may lead to concomitant changes in cell proliferation. Consistent with this notion, ZNF32 H^179^A and H^183^A significantly increased the numbers of EdU positive cells (Figure 4A, B), but there was no statistical difference between the ZNF32 H^179, 183^A and WT groups (Figure 4A, B). In addition, the results of the MTT assay (Figure 4C) and crystal violet staining (Figure 4D, E) were consistent with the EdU staining results. The above findings suggest that ZNF32 single-site mutations (H^179^A, H^183^A) can promote the proliferation of breast cancer cells.

### 2.5 ZNF32 H^179^A and H^183^A promote tumor formation and growth *in vivo*

**FIGURE 5:**
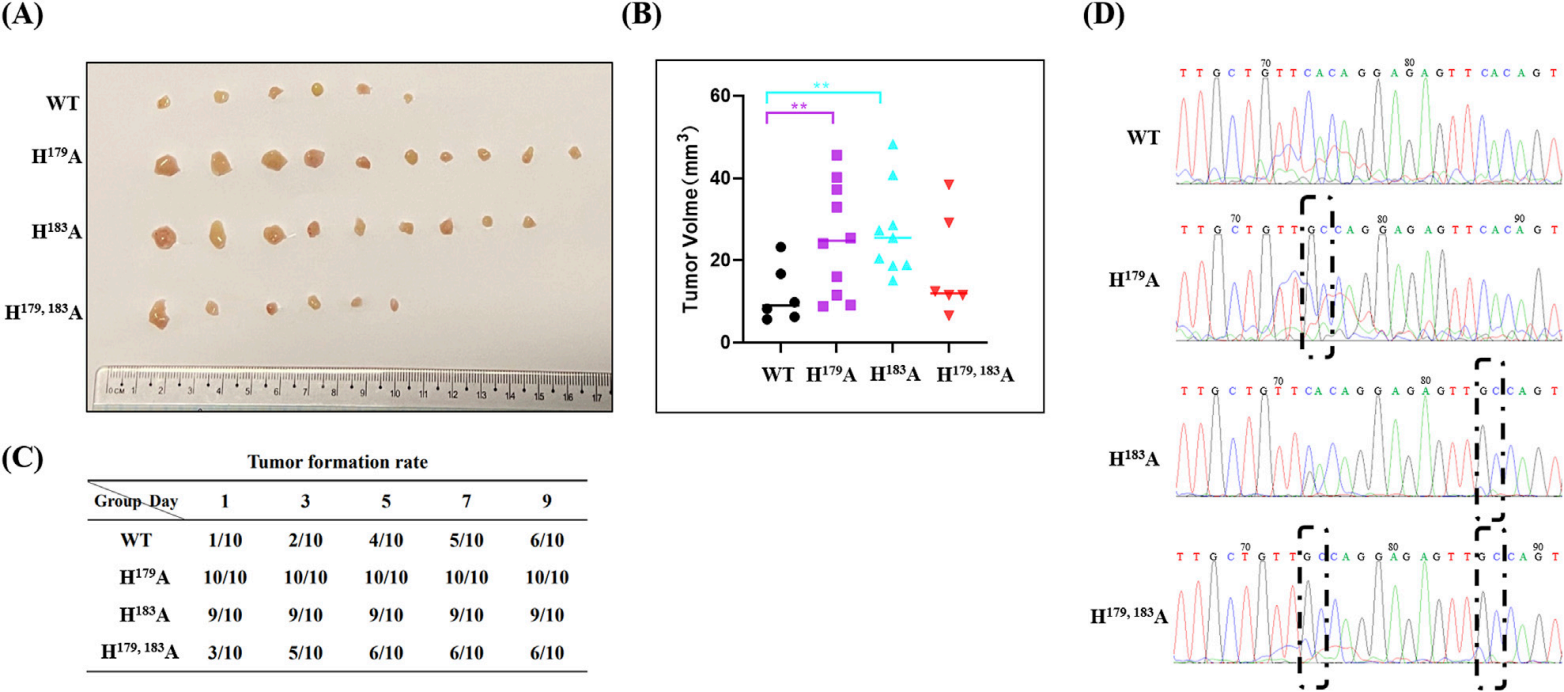
Roles of ZNF32 H^179^A, H^183^A, and H^179, 183^A in tumor formation in xenografts. A total of 1 × 10^7^ viable cells were implanted subcutaneously into nude mice. Seven days after inoculation, the mice received a vehicle. **(A)** Tumors collected 3 weeks after implantation. **(B)** Tumor volume calculations. **(C)** A tumor diameter of 3 mm was considered to be successful tumor formation, and the tumor formation durations were recorded. **(D)** Sequencing and sequence alignment of the extracted tumor samples.

To verify the consistency of the differential regulation of tumor cell proliferation by ZNF32 histidine 179 and 183 single-site and double-site mutations *in vitro* and *in vivo*, we constructed a subcutaneous xenograft tumor model in nude mice. Compared with the WT group, the tumor volumes in the ZNF32 histidine 179 and 183 single-site mutation groups were significantly higher while no significant change was noted in the ZNF32 H^179, 183^A group (Figure 5A, B). Similarly, the tumor formation rates were higher in the ZNF32 H^179^A and H^183^A groups but not significant in the ZNF32 H^179, 183^A group compared to the WT group (Figure 5C). Consistent with these results, the mRNA extracted from the above tumor tissues were used as the template for PCR amplification, and the product sequencing results showed that the corresponding site mutations of ZNF32 histidine were indeed present in the tumor cells (Figure 5D). Overall, these data indicate that ZNF32 histidine 179 and 183 single-site mutations can promote tumor growth *in vivo*.

### 2.6 NSs resulting from ZNF32 H^179, 183^A, H^179^A, and H^183^A differentially regulate breast cancer cell proliferations by differentially targeting *ISY1-RAB43*, *UPK3BL1*, and *SNX22* expressions

**FIGURE 6:**
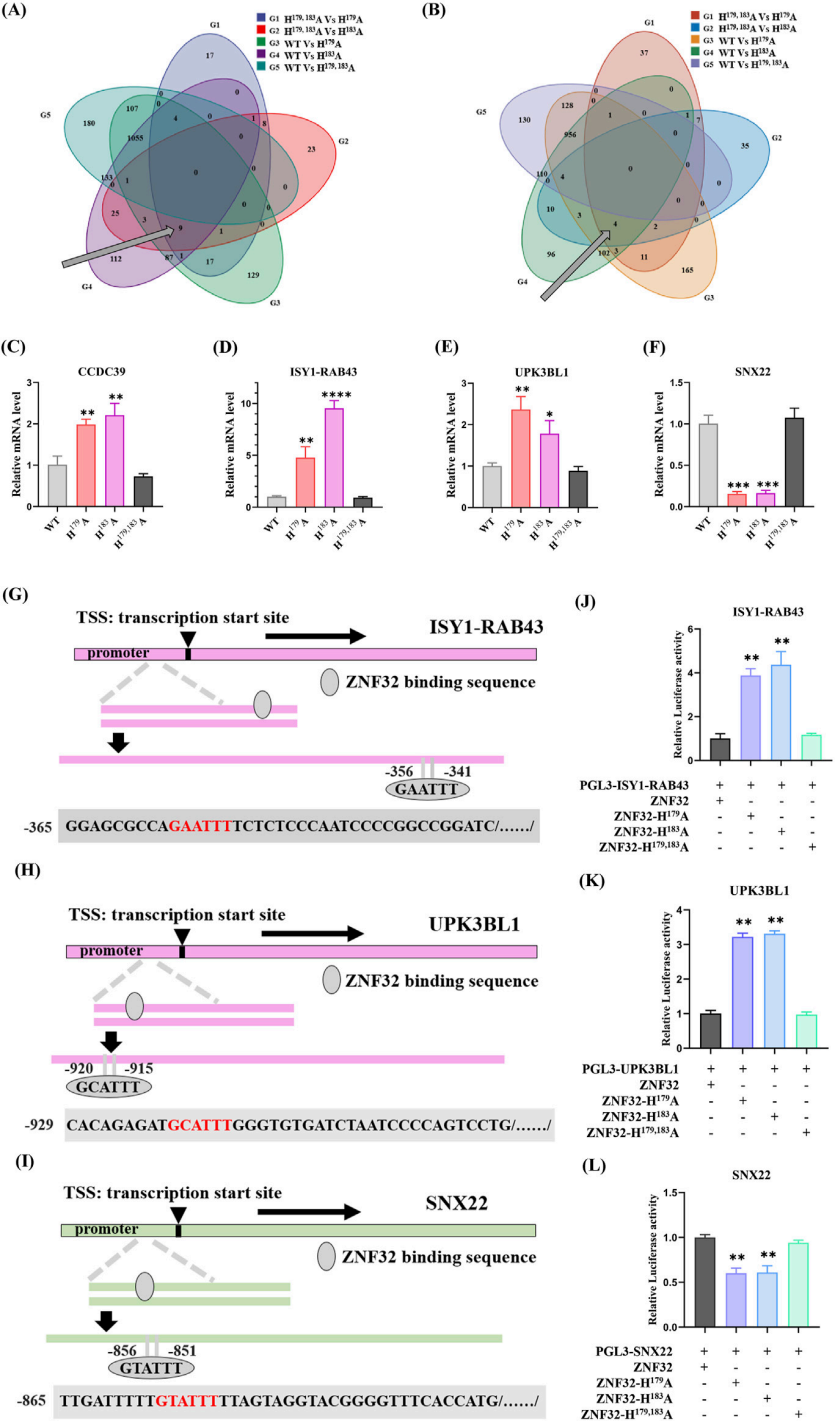
ZNF32 H^179^A and H^183^A differentially regulate breast cancer cell proliferation by differentially targeting *ISY1-RAB43*, *UPK3BL1*, and *SNX22* expressions. Venn diagrams of the **(A)** upregulated and **(B)** downregulated DEGs. RT-qPCR analysis results showing the relative expressions of **(C)** *CCDC39*, **(D)** *ISY1-RAB43*, **(E)** *UPK3BL1*, and **(F)** *SNX22*. ZNF32-binding sequences predicted in the promoter regions of **(G)** *ISY1-RAB43*, **(H)** *UPK3BL1*, and **(I)** *SNX22*. Dual luciferase reporter assay results of the targeting relationships between ZNF32 and **(J)** *ISY1-RAB43*, **(K)** *UPK3BL1*, and **(L)** *SNX22*. *p < 0.05, **p < 0.01.

As shown above, ZNF32 H^179^A, H^183^A, and H^179, 183^A differentially regulate breast cancer cell proliferation *in vivo* and *in vitro*, and we explored whether the single-site mutations could regulate proliferation through specific regulation of the downstream gene expressions. We then analyzed the transcriptome sequencing data and found the DEGs in the single-site mutation groups to explain the stronger proliferation abilities of cancer cells versus the WT and H^179, 183^A groups. According to our analysis results, there were nine upregulated (Figure 6A) and four downregulated (Figure 6B) differential genes. After removing the new and low-expression genes, three upregulated differential genes were found to be related to cell proliferation, namely *CCDC39*, *ISY1-RAB43*, and *UPK3BL1*, while *SNX22* was the only downregulated DEG. We present the differential expressions of these genes for the different groups using Log2FC values, as shown in Table 1. Then, we used RT-qPCR to detect the relative expressions of the four DEGs and found that *CCDC39*, *ISY1-RAB43*, and *UPK3BL1* expressions in the ZNF32 H^179^A and H^183^A groups were significantly higher than those in the WT and ZNF32 H^179, 183^A groups, but there were no obvious differences between the WT and ZNF32 H^179, 183^A groups (Figure 6C–E). The expressions of *SNX22* in the ZNF32 H^179^A and H^183^A cells were significantly lower than those in the WT and ZNF32 H^179, 183^A groups, and there were no obvious differences between the WT and ZNF32 H^179, 183^A cells (Figure 6F). The potential transcriptional binding sequences of ZNF32 were found in the promoter areas of *UPK3BL1*, *ISY1-RAB43*, and *SNX22* (Figure 6G–I). The dual luciferase reporter gene assay confirmed that ZNF32 H^179^A and H^183^A can transcriptionally activate *ISY1-RAB43* and *UPK3BL1* expressions while inhibiting the transcription of *SNX22* (Figure 6J–L). Together, ZNF32 H^179^A and H^183^A promote the proliferation of breast cancer cells by differentially upregulating *ISY1-RAB43* and *UPK3BL1* as well as downregulating *SNX22* expressions*.*

**TABLE 1:** Fold changes of *CCDC39*, *ISY1-RAB43*, *UPK3BL1*, and *SNX22* in different groups of cells.

#ID	Gene name	WT vs. H^179^A Log2FC	WT vs. H^183^A Log2FC	H^179,183^A vs. H^179^A Log2FC	H^179,183^A vs. H^183^A Log2FC
ENSG00000145075	*CCDC39*	10.63	10.46	10.73	10.54
ENSG00000261796	*ISY1-RAB43*	10.71	12.07	10.81	12.16
ENSG00000272949	*UPK3BL1*	7.69	7.48	4.34	4.11
ENSG00000157734	*SNX22*	−2.26	−2.49	−1.88	−2.13

### 2.7 ZNF32 H^179^A, H^183^A, and H^179, 183^A differentially regulated proliferation-related gene expressions may be related to loss of imidazole in the zinc finger protein structure

**FIGURE 7:**
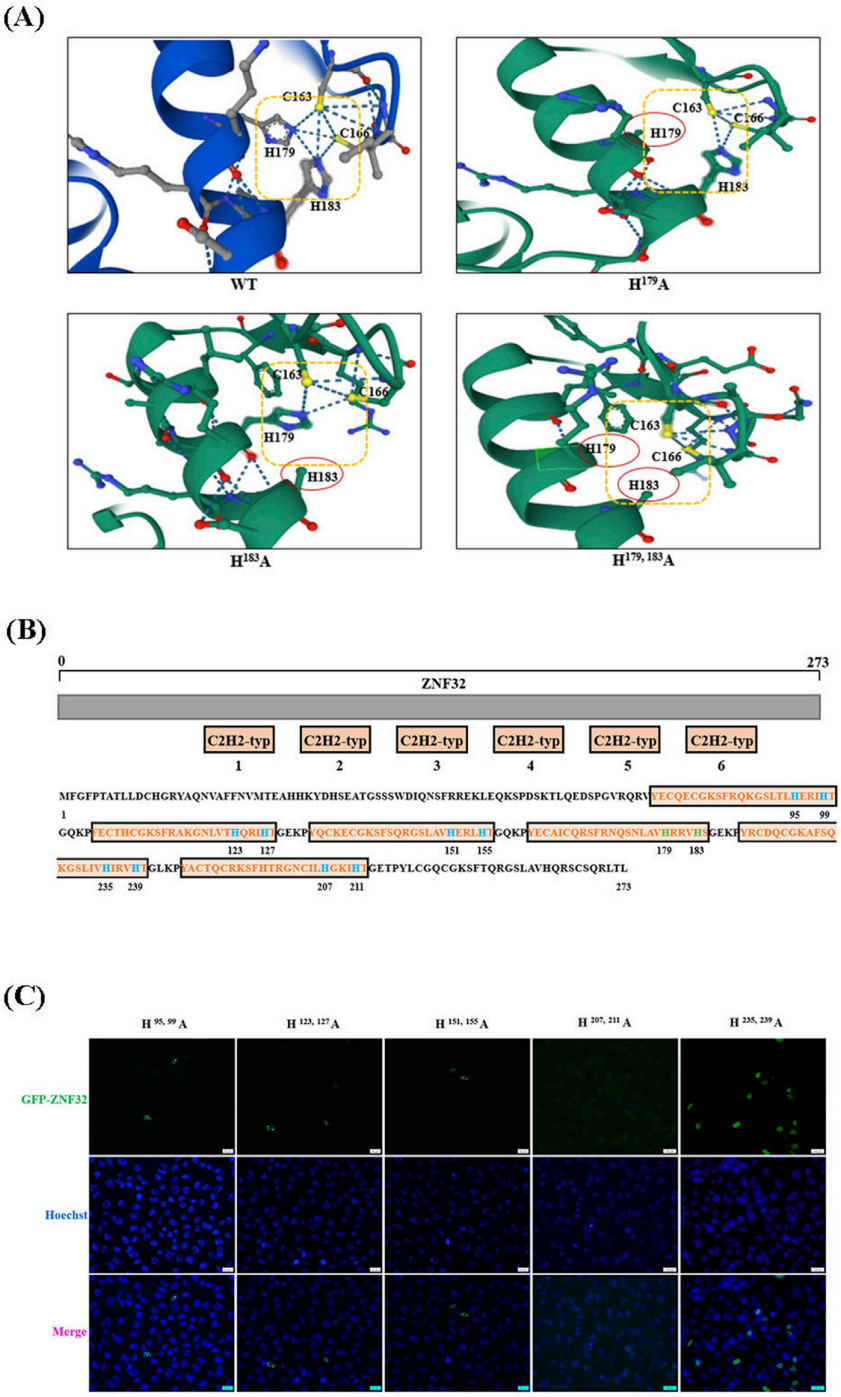
Changes in the zinc finger structures of ZNF32 can influence the proliferation of breast cancer cells and formation of nuclear speckles. **(A)** Analysis of the protein tertiary structures of ZNF32 (WT, H^179^A, H^183^A, and H^179, 183^A). **(B)** Amino acid sequence and mutation site analysis of ZNF32. **(C)** Subcellular localizations of the ZNF32 mutants (H^95, 99^A, H^123, 127^A, H^151, 155^A, H^207, 211^A, and H^235, 239^A) in ZR-75-30 breast cancer cells. The recombinant proteins are shown in green (GFP), and the cell nuclei are shown in blue (Hoechst). Scale bar = 20 μm.

As mentioned above, both single-site and double-site mutations of ZNF32 could form NSs, but the single-site mutations promote proliferation of breast cancer cells by up-down-regulating the expressions of specific genes while the double-site mutation does not affect cell proliferation. Therefore, we consider that the protein structures of ZNF32 H^179, 183^A as well as H^179^A and H^183^A could be inconsistent, thereby showing opposite regulation effects on the genes. As shown in Figure 7A, the mutation of histidine at Aa179 or Aa183 results in the loss of an imidazole ring in the zinc finger, while simultaneous mutations at these two sites can cause the loss of both imidazole rings (Figure 7A). ZNF32 has six typical C_2_H_2_-ZF domains, and the histone of the fourth zinc finger is situated at the 179 and 183 positions (Figure 7B). Therefore, we speculate that ZNF32 NS formation is largely related to the loss of the histidine imidazole ring in the zinc finger structure. Following this discovery, we mutated the histidine positions of the remaining five zinc finger structures of ZNF32 to alanine to confirm our hypothesis (Figure 7B). Interestingly, only ZNF32 H^95, 99^A, H^123, 127^A, and H^151, 155^A formed NSs in the breast cancer cells (Figure 7C), whereas H^207, 211^A showed no effect on ZNF32 localization and H^235, 239^A promoted ZNF32 to shift from nuclear to diffuse localization of the cytoplasm (Figure 7C). We conducted the same experiments on the MCF-7 and MDA-MB-231 breast cancer cell lines, whose results showed that the double-site mutation of H^207, 211^A had no effect on the localization of ZNF32 and that histidine double-site mutations at all the other zinc fingers formed NSs (Supplementary Figure S3). Thus, the different zinc finger structure mutations of ZNF32 show inconsistent formation of NS-like structures and may also have different effects on the proliferation of breast cancer cells.

## 3 Discussion

Early studies have shown clusters of hyperphosphorylated Pol II and BrU labeled transcripts associated with NSs (Wei et al., 1999). A subpopulation of the largest subunit of RNA polymerase II is located at the 20–50 discrete subnuclear domains that are closely linked to speckle formation (Bregman et al., 1995). Recent studies have shown that NSs are associated with high-level-transcribed gene-rich chromosomal domains (Hu et al., 2019; Chen and Belmont, 2019). In the present study, we found that ZNF32 H^179^A, H^183^A, and H^179, 183^A could lead to NS formation in breast cancer cells, so we performed transcriptome sequencing and non-targeted metabolomics sequencing on WT and mutant breast cancer cells. The transcriptome analysis indicated that the DEGs were mainly enriched in terms of molecular functions, such as growth factor activity, calcium binding, ATP enzyme activity, transcriptional activator activity, and RNA polymerase II transcription factor activity. Increasing evidence suggests that speckles coordinate the transcription, processing, and export of highly expressed mRNAs (Hu et al., 2019; Chen and Belmont, 2019). Studies have shown that the NSs are regions that can enhance gene expressions; they can also be used as storage and recycling sites for the splicing factors returned from splicing activities. The NSs may regulate the release of splicing factors back into the nucleoplasm, thus controlling the level of gene expression (Faber et al., 2022). The dynamic changes in the NSs depend on many factors, including cellular ATP levels, phosphorylation statuses of various proteins, transcription of stress-activated genes, SWI/SNF chromatin remodeling, as well as RNA polymerase II transcription and splicing (Faber et al., 2022; Misteli, 2007). Studies have shown that proteins involved in chromosome mapping, chromatin modification, transcription, splicing, 3′-terminal processing, mRNA modification, mRNA-coated proteins, and messenger ribonucleoprotein (mRNP) output are assembled in the NSs. Importantly, all of these steps are coupled to the transcription of RNA polymerase II, which occurs in the chromatin fibrils near the NSs. Similarly, our transcriptome sequencing results show that the transcriptional activity of RNA polymerase II is important for NS formation. At present, there is very sparse research on NSs , and the specific reasons and mechanisms of NS formation need to be explored through further experiments.

NSs were initially considered as sites for storing and modifying splicing factors, but they are now recognized as nucleosomes that promote comprehensive regulation of gene expressions. In addition, we found that NS formation is closely related to the signaling pathways of cancer cell proliferation, such as MAPK, PI3K-Akt, Rap1, and RAS. The MAPK and PI3K-Akt pathways have been reported to play key roles in cell proliferation, differentiation, and death (Yang et al., 2003; Asl et al., 2021; Yu and Cui, 2016). Mutations of key molecules involved in the signal transduction and dysregulation of the MAPK pathway can affect tumor growth, apoptosis, angiogenesis, invasion, metastasis, and drug resistance (Sun et al., 2015; Fang and Richardson, 2005; Li et al., 2024; Pan et al., 2023). Rap1 is a member of the Ras small GTP family and is activated by many extracellular stimuli, including growth factors, cytokines, as well as intercellular and extracellular matrix adhesions (Stork, 2003; Bos, 2005); its biological functions seem to be very complex, ranging from inhibiting or stimulating cell growth and differentiation (Pan et al., 2018) to even promoting the adhesion, migration, and invasion of cancer cells (Wei et al., 2023). Overexpression of Rap1 has been reported to induce carcinogenic transformations in cultured fibroblasts (Altschuler and Ribeiro-Neto, 1998). GTP enzymes of the Ras family transduce signals from various receptors, including receptor tyrosine kinases, G-protein-coupled receptors, and cytokine receptors, to regulate various signal pathways to promote cell proliferation, cell survival, and gene expression (Vigil et al., 2010; Rojas et al., 2011). RAS was the first oncogene discovered in human cancer cells, and researchers have since discovered a wide range of RAS mutations in human patient samples (Baines et al., 2011). Therefore, the mutated Ras protein plays a key role in tumorigenesis and maintenance (Chin et al., 1999). Comprehensive transcriptomics and metabolomics analyses revealed that ZNF32 NS formation was accompanied by changes in choline metabolism. Compared with normal cells, cancer cells require metabolic reprogramming to support their high proliferation rates and survival (Glunde and Serkova, 2006; Jia et al., 2016). Abnormal choline metabolism has emerged as a metabolic hallmark associated with tumorigenesis and tumor progression (Bagnoli et al., 2016); it reflects the complex interplay between oncogenic signaling and cellular metabolism (Glunde et al., 2011). Among the DEMs that we enriched in this work, the ZNF32 mutant cells showed lower levels of lysophosphatidylcholine [LysoPC (15:0), LysoPC (16:0), and LysoPC (17:0)] than WT cells. LysoPC is a hemolytic lipid produced by the oxidation of low-density lipoproteins, and its known functions include immune regulation, apoptosis induction, oxidative stress, and anti-infection activity (Liu et al., 2020). Recently, researchers reported that LysoPC could be a tumor marker, where low levels of LysoPC (16:0) are associated with the occurrences of various cancers, including colorectal cancer, intrahepatic bile-duct carcinoma, and ovarian cancer (Zhao et al., 2007; Kim et al., 2017; Kim et al., 2014); LysoPC (17:0) is also considered as a biomarker for hepatocellular carcinoma (HCC) (Ressom et al., 2012). The study also reported that LysoPC inhibits the adhesion and metastasis of cancer cells by changing the morphology of the tumor cell membranes (Mahadeo and Prenner, 2020). In addition, LysoPC reduction has been observed in patients with advanced lung and prostate cancers as well as cancer metastasis (Zhu et al., 2020; Goto et al., 2015). Therefore, our results indicate that NS formation is closely related to changes in the above three LysoPC levels. However, the specific mechanism of NS formation and its connection with the choline metabolic pathway require further study.

Based on the results of the combined transcriptome and metabolome sequencing analyses, we explored the effects of ZNF32 H^179^A, H^183^A, and H^179, 183^A on breast cancer cells. The *in vivo* and *in vitro* experiments showed that ZNF32 H^179^A and H^183^A promote proliferation of breast cancer cells. The DEGs *CCDC39*, *ISY1-RAB43*, *UPK3BL1*, and *SNX22* result in strong proliferation ability of ZNF32 H^179^A and H^183^A cells. Some studies showed that *CCDC39* mutations in cells showed higher levels of proinflammatory cytokines (Varenyiova et al., 2023); in addition, *CCDC39* and *SNX22* are closely related to the growth and development of mammals (Abdelhamed et al., 2018; Hu et al., 2022). *UPK3BL1* is reportedly related to the lipopolysaccharide-induced apoptosis of nucleus pulposus cells (Zhang et al., 2020). Studies have shown that pre-mRNA splicing factor 1 homologs (ISY1) are upregulated at both the transcriptomic and proteomic levels in the initiation, progression, and tumor stages of HCC (Shaglouf et al., 2023). Because ZNF32 H^179^A and H^183^A promote the proliferation of breast cancer cells while ZNF32 H^179, 183^A does not, the protein structure analysis showed that the structures of H^179, 183^A, H^179^A, and H^183^A of ZNF32 were different. We hypothesize that changes in the protein structure caused these genes to show opposing regulatory effects. In addition, protein sequence analysis showed that the mutated histidine was located in the typical C_2_H_2_-ZF structure, proving that such a mutation would destroy the zinc finger structure and form NSs. Subsequent mutations of histidine in the other zinc finger structures also confirmed this assumption. However, the roles and mechanisms of the aforementioned genes in the proliferation of breast cancer cells as well as the molecular mechanism of regulation of NS formation by the zinc finger structures need to be studied further.

## 4 Conclusion

**FIGURE 8:**
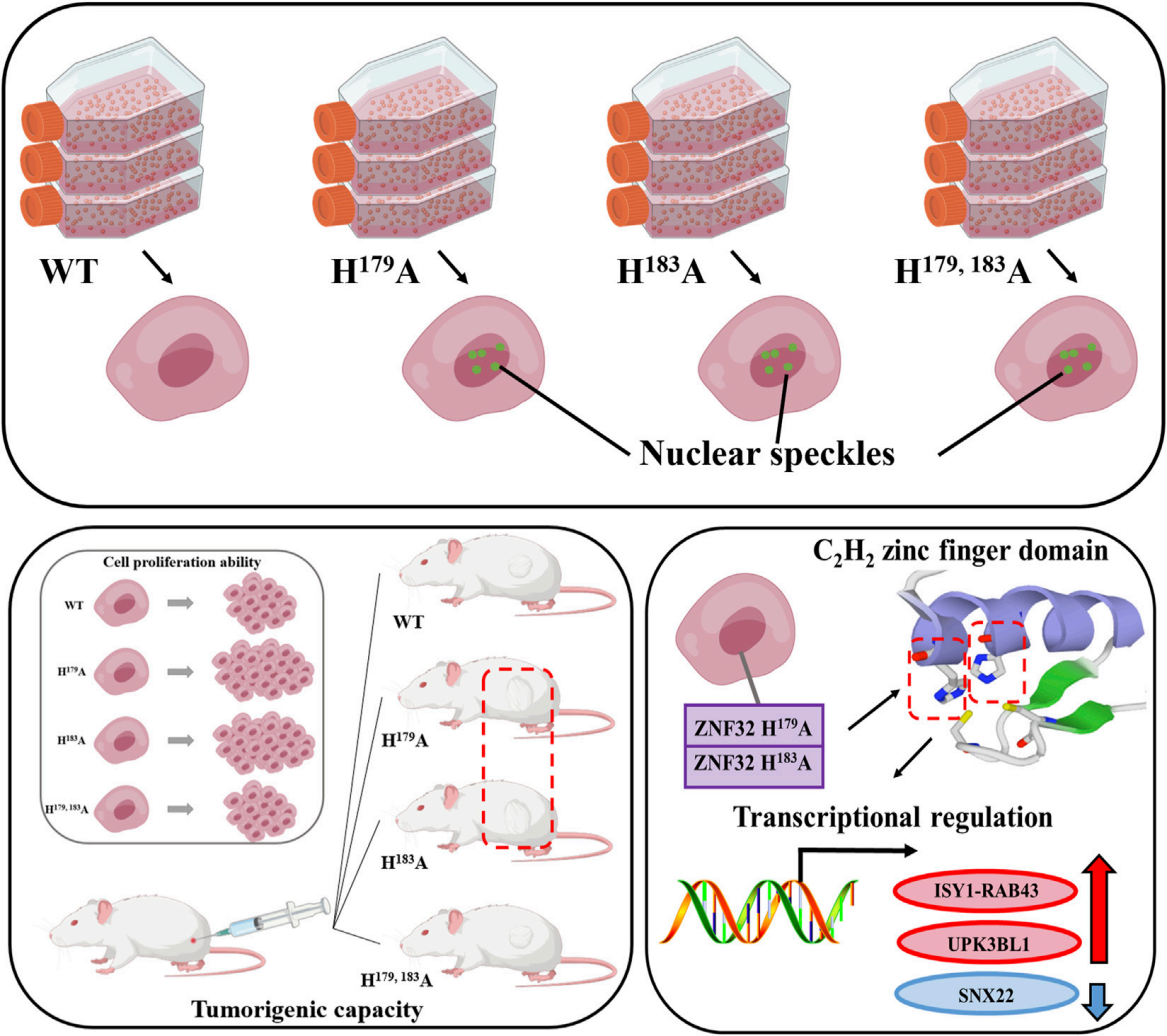
Effects and mechanisms of ZNF32 histidine 179 and 183 single-site and double-site mutations on breast cancer cells. Both single-site and double-site mutations promote formation of nuclear speckles of this protein but differentially regulate the proliferation of breast cancer cells.

In this study, we validated that ZNF32 H^179^A, H^183^A, and H^179, 183^A promote NS formation; however, *in vitro* and *in vivo* experiments suggest that only ZNF32 H^179^A and H^183^A promote the proliferation of breast cancer cells through the loss of one imidazole ring on the fourth zinc finger structure as well as differential upregulation of *ISY1-RAB43* and *UPK3BL1* along with downregulation of *SNX22* expressions (Figure 8). This study deepens the understanding of the functions of ZNF32 mutants and NSs in breast cancer cells while providing a basis for exploring novel treatments for breast cancer.

## 5 Materials and methods

### 5.1 Cell culture

The human breast cancer cell lines ZR-75-30, MCF-7, and MDA-MB-231 were obtained from the American Type Culture Collection (Manassas, VA, United States) and maintained in RPMI 1640 medium containing 10% fetal bovine serum (FBS; Gibco, United States) in a humidified atmosphere at 37°C and 5% CO_2_ as well as tested regularly for *mycoplasma* to verify its negative status.

### 5.2 Construction of stable ZNF32 H^179, 183^A, H^179^A, and H^183^A breast cancer cell lines

ZNF32 overexpressed (WT) and mutated (H^179, 183^A, H^179^A, and H^183^A) lentivirus samples were purchased from Genepharm (Shanghai, China). All procedures were performed as per the manufacturer instructions. The stable mutated (ZNF32 H^179, 183^A, H^179^A, and H^183^A) and WT cell lines were selected with puromycin.

### 5.3 Construction of vectors and transfection

GFP-ZNF32 plasmids were constructed and stored in our lab. The primers used are listed in Table 2, and all mutations were generated using Mut Express II Fast Mutagenesis Kit V2. (#C214-01 from Vazyme, Nanjing, China). Sequences containing promoter binding regions of ISY1-RAB43, UPK3BL1, and SNX22 were synthesized and constructed into PGL3-Basic vectors by NheI/HindIII (#CD01975871, #CD01975872, #CD01975873 from Tsingke Biotech, Beijing, China). All transfection experiments were performed with TurboFect Transfection Reagent (Thermo, Waltham, MA, United States) according to the manufacturer’s instructions.

**TABLE 2:** Primers for ZNF32 plasmid construction.

Primer name	Primer sequence
GFP-N1-H^179,183^A-up	GAG​AGT​TGC​CAG​TGG​TGA​GAA​GCC​CTA​TAG​ATG​T
GFP-N1-H^179,183^A -down	CCA​CTG​GCA​ACT​CTC​CTG​GCA​ACA​GCA​AGG
GFP-N1-H^179^ A-up	ATC​AGA​GTA​ACC​TTG​CTG​TTG​CCA​GGA​GAG​TT
GFP-N1-H^179^A -down	GCA​ACA​GCA​AGG​TTA​CTC​TGA​TTC​CTG​AAG
GFP-N1-H^183^A-up	TTG​CTG​TTC​ACA​GGA​GAG​TTG​CCA​GTG​GTG​AG
GFP-N1-H^183^A -down	GCA​ACT​CTC​CTG​TGA​ACA​GCA​AGG​TTA​CTC​TG
GFP-N1-H^95,99^A-up	GAG​AAT​CGC​CAC​TGG​TCA​AAA​GCC​TTT​TGA​GTG​C
GFP-N1-H^95,99^A -down	CCA​GTG​GCG​ATT​CTC​TCA​GCT​AAC​GTT​AGA​C
GFP-N1-H^123,127^A-up	ACG​GAT​AGC​CAC​GGG​AGA​GAA​GCC​TTA​TCA​GTG
GFP-N1-H^123,127^A -down	CCC​GTG​GCT​ATC​CGT​TGA​GCT​GTA​ACA​AGA​TTG
GFP-N1-H^151,155^A-up	GAG​ACT​CGC​CAC​TGG​ACA​GAA​ACC​CTA​CG
GFP-N1-H^151,155^A -down	CCA​GTG​GCG​AGT​CTC​TCG​GCG​ACA​GC
GFP-N1-H^235,239^A-up	CAA​AAT​CGC​CAC​AGG​AGA​GAC​ACC​CTA​TCT​GTG
GFP-N1-H^235,239^A -down	CCT​GTG​GCG​ATT​TTG​CCA​GCC​AGA​ATA​CAA​TTC
GFP-N1-H^207,211^A-up	CAG​AGT​CGC​CAC​AGG​CCT​GAA​GCC​CTA​TGC
GFP-N1-H^207,211^A -down	CCT​GTG​GCG​ACT​CTG​ATG​GCA​ACA​ATT​AAG​CT

### 5.4 EdU staining

For the 5-ethynyl-20-deoxyuridine (EdU) assay, the 5-8F-kiss1R and 5-8F-vehicle cells were seeded in 24-well plates at a density of 1 × 10^5^ cells/well, and the assay was carried out according to the instructions on the kit (Beyotime). The cells were transfected with kiss1 and control plasmids for 48 h as well as incubated for 2 h in a preheated EdU working solution (10 M) at 37°C. The cells were then washed thrice, and a permeable solution was added to the 24-well plates for incubation for 10–15 min. After washing three more times, approximately 200 μL of the click reaction mixture was added and the cells were incubated in the dark at room temperature for 30 min. The samples were then washed thrice, the cell nuclei were stained with DAPI for 5 min, and the cells were finally washed thrice with phosphate-buffered saline (PBS); the prepared cells were then observed and imaged with a microscope.

### 5.5 MTT assay

The MTT assay was performed as per manufacturer protocols using the MTT Cell Viability Assay Kit (Biotechwell WH1197). The cells were seeded in 96-well plates at 10^4^ cells/well and construct transfected 20 h post seeding, as indicated by the manufacturer. MTT was subsequently added to the culture medium and incubated for 2 h at 37°C. Then, the medium was discarded, and 150 μL of dimethylsulfoxide (DMSO) was added to each well. The absorbances of the samples were then measured at 490 nm.

### 5.6 Crystal violet staining

The cells were seeded in 6-well plates and cultured for 2 weeks; during the incubation process, the medium was changed every 24 h. The cell colonies were fixed and stained using a buffer containing 0.05% w/v crystal violet, 1% formaldehyde, and 1% methanol in 1 × PBS at 20°C for 30 min. The samples were then thoroughly washed using ddH_2_O and air-dried as per manufacturer protocols.

### 5.7 Animals

Six-week-old BALB/c female nude mice (Dashuo, Chengdu, China) were used in this study. About 5 × 10^6^ viable ZR-75-30 cells with the ZNF32 mutation, including WT, H^179, 183^A, H^179^A, and H^183^A, were subcutaneously injected into the mice. One week following subcutaneous transplantation, we observed and recorded the tumor growth and formation rates. The Institutional Animal Care and Use Committee of Southwest Minzu University (Chengdu, China) approved this research project, and all animal experiments were conducted in line with the animal ethical treatment protocols.

### 5.8 RNA extraction, quality control, and sequencing

When the cells were cultured to 90% in a 10-cm culture dish, the consumed culture medium was discarded, cells were washed twice with PBS before adding TRlzol for lysis, and lysed cells were transferred to an Eppendorf tube. The total RNA was extracted in accordance with the instruction manual of the TRlzol Reagent (Life Technologies, CA, United States). The concentration and purity of the RNA were measured using the NanoDrop 2000 (Thermo Fisher Scientific, Wilmington, United States), and the Agilent Bioanalyzer 2100 system (Agilent Technologies, CA, United States) was used to evaluate RNA integrity. The sequencing library was successfully constructed using the Hieff NGS Ultima Dual-Mode mRNA Library Prep Kit for Illumina (Yeasen Biotechnology, Shanghai, China). The quality of the library was evaluated using the AMPure XP system, and the library was sequenced on the Illumina NovaSeq platform to produce a double-ended read length of 150 bp. The sequencing data generated in this study have been deposited in the NCBI SRA database under the bioproject number PRJNA1135469 (https://www.ncbi.nlm.nih.gov/bioproject/?term=%20PRJNA1135469).

### 5.9 Differential expression quantification and analysis

The expression level of each gene was normalized with the reads per kilobase per million (RPKM). To identify the DEGs, the edgeR package was used to filter the genes (Robinson et al., 2010). Following statistical analyses, we screened the DEGs with fold changes ≥3 by setting the false discovery rate (FDR) to <0.01.

### 5.10 GO and pathway analyses

GO enrichment analysis of the DEGs was performed using the GOseq R package (Young et al., 2010). All identified DEGs were then annotated using the KEGG database (Kanehisa et al., 2008). Additionally, a hypergeometric test was conducted to find the pathways that were significantly enriched in terms of the DEGs compared to the whole-genome background.

### 5.11 Metabolite extraction

In this study, using the liquid chromatography quadrupole-time-of-flight (LC-QTOF) platform, a total of 24 samples from the mutation groups (ZNF32 H^179, 183^A, H^179^A, and H^183^A) and WT group corresponding to six samples from each group were subjected to qualitative and quantitative metabolome analyses. The liquid chromatography mass spectrometry (LC/MS) system used for metabolomics analysis was composed of the Waters Acquity I-Class PLUS ultrahigh-performance liquid tandem Waters Xevo G2-XS QTOF high-resolution mass spectrometer. The column used was the Waters Acquity UPLC HSS T3 (1.8 μm, 2.1 × 100 mm). The positive and negative ion modes were both composed of 0.1% formic acid aqueous solution as mobile phase A and 0.1% formic acid acetonitrile as mobile phase B, with an injection volume of 1 μL.

### 5.12 LC-MS/MS analysis

The Waters Xevo G2-XS QTOF high-resolution mass spectrometer was used to collect primary and secondary MS data in the MSe mode using the MassLynx V4.2 (Waters) acquisition software. During each data acquisition cycle, dual-channel data acquisition was performed using both low and high collision energies at the same time. The low collision energy used was 2 V, while the high collision energy range was 10–40 V, with a scanning frequency of 0.2 s for a mass spectrum. The parameters of the electrospray ionization (ESI) source are as follows: capillary voltage of 2,000 V (positive ion mode) or −1,500 V (negative ion mode); cone voltage of 30V; ion source temperature of 150°C; desolvent gas temperature of 500°C; backflush gas flow rate of 50 L/h; desolvent gas flow rate of 800 L/h.

### 5.13 Data preprocessing and annotation

The raw data collected using MassLynx V4.2 was processed for peak extraction, peak alignment, and other data processing operations based on the Progenesis QI software online METLIN database and Biomark’s self-built library for identification; at this time, the theoretical fragment identification and mass deviation were both within 100 ppm.

### 5.14 Metabolomics analysis

A follow-up analysis was performed after normalizing the original peak area information with the total peak area. Principal component analysis and Spearman correlation analysis were used to assess the repeatability of the samples within the group and quality control samples. The identified compounds were searched for classification and pathway information in the KEGG, HMDB, and LIPID MAPS databases. Based on the grouping information, we calculated and compared the difference multiples. The R language package ropls was used to perform orthogonal partial-least-squares discriminant analysis (OPLS-DA) modeling, and 200-factor permutation tests was performed to verify the model reliability. The variable importance in projection (VIP) value of the model was calculated using multiple cross-validations. The difference multiple, *p* value, and VIP value of the OPLS-DA model were combined to screen the differential metabolites with thresholds of VIP ≥1 and fold change ≥1. The difference metabolites of the KEGG pathway enrichment significance were calculated using the hypergeometric distribution test.

### 5.15 RT-qPCR validation

To validate the DEGs discovered by transcriptome sequencing, RT-qPCR was performed. Primer Premier 5 software was used to design the sequence-specific primers for the selected genes (Table 3). Thereafter, RT-qPCR was performed with the qPCR SYBR Green SuperMix according to manufacturer instructions (Bimake, United States). The 18S rRNA gene was used as the endogenous reference to normalize the relative mRNA expression.

**TABLE 3:** Primers for the RT-qPCR analysis in this study.

Primer name	Primer sequence
18S-up	TTG​ACG​GAA​GGG​CAC​CAC​CAG
18S-down	GCA​CCA​CCA​CCA​CGG​AAT​CG
SNX22-up	AAT​TCC​TGA​GAC​TTC​GGC​ACT​TCC
SNX22-down	GGA​GCA​CAC​CAT​TCA​CCA​CCA​C
CCDC39-up	ATA​CAC​AGC​AAT​GGA​AGA​GCG​AAC​T
CCDC39-down	GGA​GGC​AGC​ATA​ACA​ACA​GTC​AGA​A
ISY1-RAB43-up	CCC​TCG​CAG​CAA​GAG​ATT​GA
ISY1-RAB43-down	CCA​TTG​GCA​CTG​CGG​TAG​TA
UPK3BL1 -up	CCA​GCT​CTC​AAA​CGA​CAC​CT
UPK3BL1 -down	AGT​AGC​CCC​TCT​GGG​AGA​AG

### 5.16 Dual-Luciferase reporter assay

ZR-75-30 cells were seeded at 20,000 cells per well in 500 μL of medium in 24-well plates for 24 h. Using 1 μg firefly luciferase report plasmid (*PGL3-ISY1-RAB43*, *PGL3-UPK3BL1*, *PGL3-SNX22*) and 1 μg ZNF32 wild type or mutants (ZNF32 H^179^A, ZNF32 H^183^A, ZNF32 H^179, 183^A) and 0.1 μg renilla plasmid pRL-TK were co-transfected into breast cancer cells. Forty-eight hours after transfection, cells were lysed and luciferase activity measured according to the manufacturer’s instructions (Dual Luciferase Reporter Assay Kit, #DL101, Vazyme, Nanjing, China). Finally, the luminescence signals of firefly luciferase and renilla luciferase were measured by a Varioskan™ LUX multimode microplate reader (#VLBLATGD2, Thermo Fisher Scientific, United States).

### 5.17 Statistical analysis

The quantitative PCR data were analyzed using the 2^-ΔΔCt^ method, and the data were expressed as mean ± standard deviation (Mean ± SD). The differences in the data were analyzed using one-way ANOVA, multiple comparison t-test, and student’s two-tailed t-test in GraphPad Prism 8.0 software. All experiments were repeated at least three times and were statistically significant when the *p* values were <0.05, *p < 0.05, **p < 0.01, ***p < 0.001, and ****p < 0.0001.

## Data Availability

The datasets presented in this study can be found in online repositories. The sequencing data generated in this study have been deposited in the NCBI SRA database (bioproject number: PRJNA1135469) and can be reviewed via the following link: https://www.ncbi.nlm.nih.gov/bioproject/?term=%20PRJNA1135469.
