# Supplementary material for: ZNF32 histidine 179 and 183 single-site and double-site mutations promote nuclear speckle formation but differentially regulate the proliferation of breast cancer cells
Source: Front Cell Dev Biol. 2025 Feb 19;13:1490231. doi: 10.3389/fcell.2025.1490231 (PMC11880268; doi:10.3389/fcell.2025.1490231)
Supplement: Supplementary file 1 [file DataSheet1.zip › Supplementary Files/Supplementary figure legends.pdf]

Supplementary figure legends

Figure S1. The subcellular localization of ZNF32 mutants ($H^{179}A$, $H^{183}A$, $H^{179, 183}A$) in different breast cancer cell lines. (A) The subcellular localization of ZNF32 mutants ($H^{179}A$, $H^{183}A$, $H^{179, 183}A$) in MCF-7 breast cancer cells. (B) The subcellular localization of ZNF32 mutants ($H^{179}A$, $H^{183}A$, $H^{179, 183}A$) in MDA-MB-231 breast cancer cells. Recombinant proteins are shown in green (GFP), and cell nucleus are shown in blue (Hoechst). Scale bar = 20 μm.

Figure S2. The relative mRNA expression of ZNF32 in ZR-75-30 cells (NC), ZR-75-30 ZNF32 overexpression cells (WT), ZR-75-30 ZNF32 $H^{179}A$, $H^{183}A$, $H^{179, 183}A$ cells. qRT-PCR analysis showed the relative expression of ZNF32 in mRNA level. $^*P < 0.05$, $^{**}P < 0.01$.

Figure S3. The subcellular localization of ZNF32 mutants ZNF32 ($H^{95, 99}A$, $H^{123, 127}A$, $H^{151, 155}A$, $H^{207, 211}A$, $H^{235, 239}A$) in different breast cancer cell lines. (A) The subcellular localization of ZNF32 mutants ZNF32 ($H^{95, 99}A$, $H^{123, 127}A$, $H^{151, 155}A$, $H^{207, 211}A$, $H^{235, 239}A$) in MCF-7 breast cancer cells. (B) The subcellular localization of ZNF32 mutants ZNF32 ($H^{95, 99}A$, $H^{123, 127}A$, $H^{151, 155}A$, $H^{207, 211}A$, $H^{235, 239}A$) in MDA-MB-231 breast cancer cells. Recombinant proteins are shown in green (GFP), and cell nuclei are shown in blue (Hoechst). Scale bar = 20 μm.
