# Supplementary figures and images for: ZNF32 histidine 179 and 183 single-site and double-site mutations promote nuclear speckle formation but differentially regulate the proliferation of breast cancer cells
Source: Front Cell Dev Biol. 2025 Feb 19;13:1490231. doi: 10.3389/fcell.2025.1490231 (PMC11880268; doi:10.3389/fcell.2025.1490231)

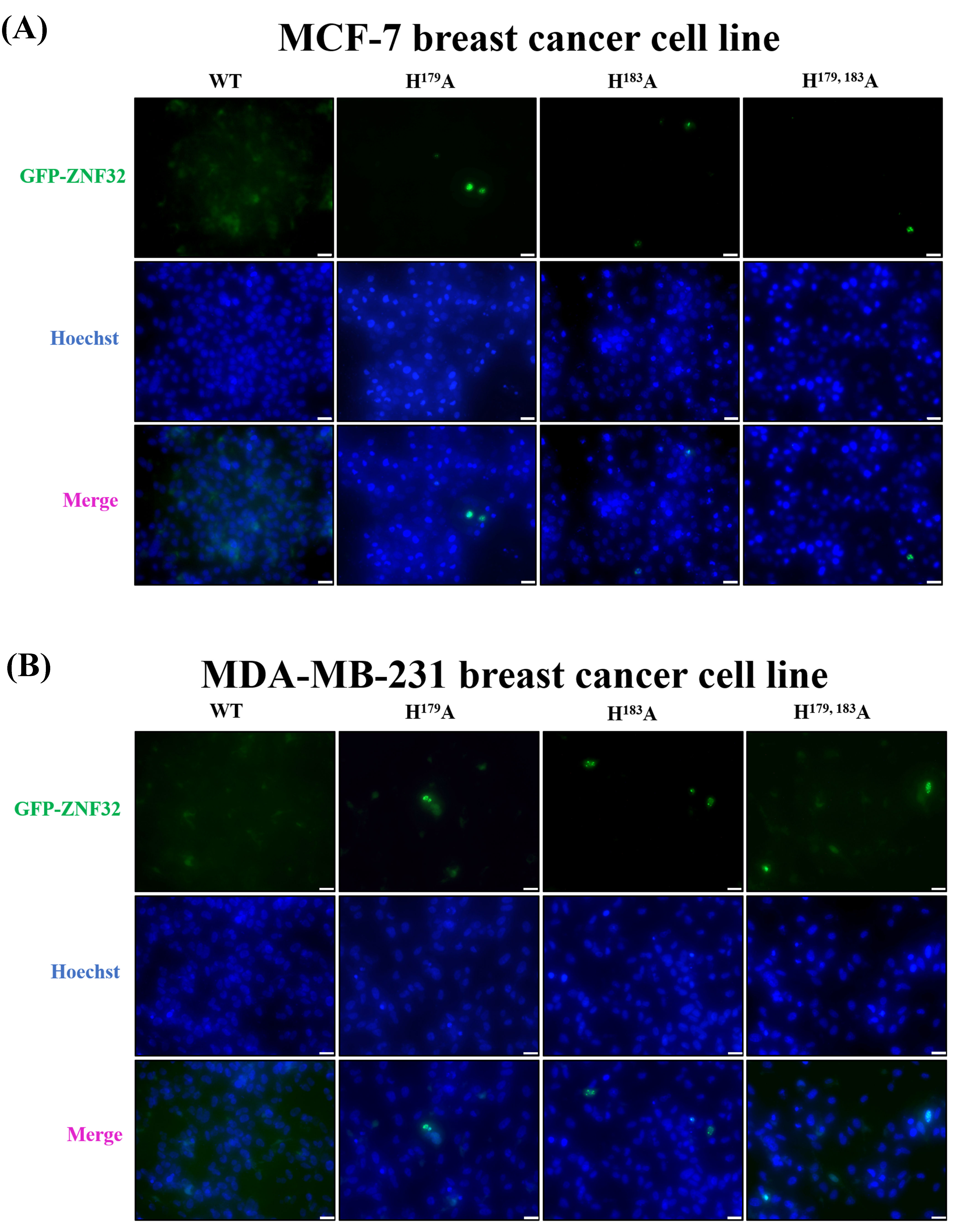

Supplement: Supplementary file 1 [file DataSheet1.zip › Supplementary Files/Figure S1.jpg]

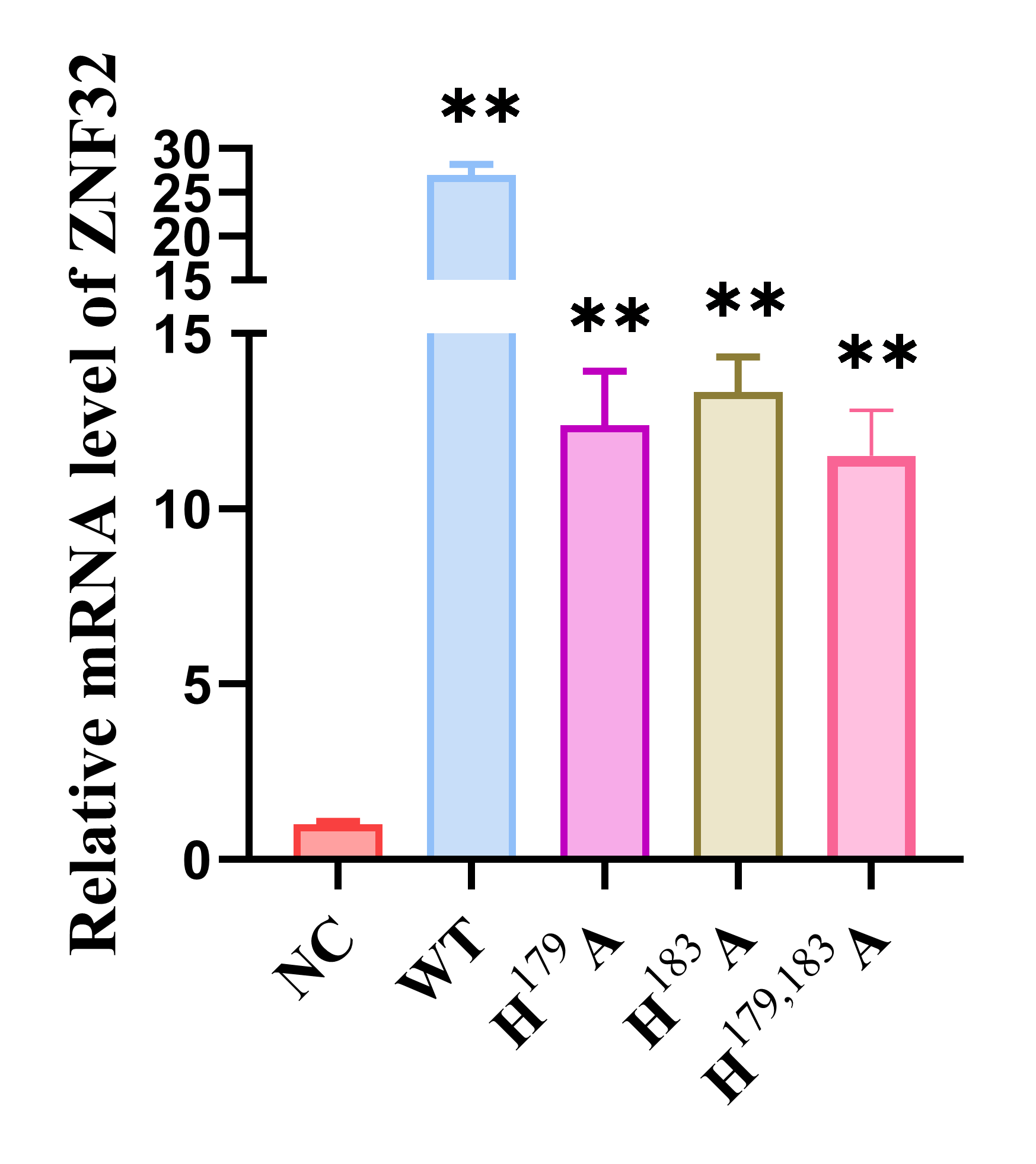

Supplement: Supplementary file 1 [file DataSheet1.zip › Supplementary Files/Figure S2.tif]
